# Comparison of microRNA expression profiles of Kashin-Beck disease, osteoarthritis and rheumatoid arthritis

**DOI:** 10.1038/s41598-017-00522-z

**Published:** 2017-04-03

**Authors:** Wenhong Wu, Awen He, Yan Wen, Xiao Xiao, Jingcan Hao, Feng Zhang, Xiong Guo

**Affiliations:** Key Laboratory of Trace Elements and Endemic Diseases of Ministry of Health, School of Public Health, Health Science Center, Xi’an Jiaotong University, Xi’an, P.R. China

## Abstract

Kashin-Beck disease (KBD) is a chronic osteochondropathy with unclear pathogeny. In this study, we compared the microRNA expression profiles of 16 KBD patients, 16 osteoarthritis (OA) patients and 16 rheumatoid arthritis (RA) patients and 16 healthy controls in their blood specimens. miRNAs expression profiling was performed using Exiqon miRCURY LNATM miRNAs Array. miRNAs target genes were predicted using miRror suite. Another independent mRNA expression profile dataset of 20 KBD patients and 15 healthy controls were integrated with the miRNA expression profiles of KBD. We identified 140 differently expressed miRNAs in KBD vs. Controls. GO enrichment analysis found that hypoxia, Wnt receptor signaling pathway and vitamin B6 biosynthesis related GO terms were significantly overrepresented in the target genes of differently expressed miRNAs in KBD vs. Control. 18 differently expressed common miRNAs were identified in KBD vs. Control, KBD vs. OA and KBD vs. RA. Integrating the lists of differently expressed miRNA target genes and mRNA differently expressed genes detected 6 common genes for KBD. Our results demonstrated the altered miRNAs expression profiles of KBD comparing to healthy controls, OA and RA, which provide novel clues for clarifying the mechanism of KBD.

## Introduction

Kashin-Beck disease (KBD) is a chronic osteochondropathy^1, 2^, which is characterized by cartilage degeneration, cartilage matrix degradation, chondrocyte necrosis and apoptosis in growth plate cartilage and articular cartilage^3, 4^. In China, there are more than 2.5 million KBD patients, and about 30 million people living in KBD prevalent areas are at the risk of KBD. Because the pathogenesis of KBD remains elusive, there is not effective curing method for KBD now. The clinical manifestations of KBD mainly include pain, limited motion and deformities of joints^5, 6^, which are similar to the clinical manifestations of osteoarthritis (OA) and rheumatoid arthritis (RA). It is often very difficult to distinguish KBD from OA and RA.

microRNAs (miRNAs) are non-coding single-stranded small RNAs, which can regulate gene expression via complementarity to the 3′-UTR of mRNAs of target genes. Recent studies found that miRNAs played an important role in the joint injuries of OA and RA. For instance, Jones S.W. *et al*. observed significant difference in miRNAs expression profiles between OA and healthy controls^7^. miRNAs-140 knock-out mice exhibited proteoglycan loss and growth retardation of articular cartilage^8^, which were the two major pathological changes of KBD articular cartilage. However, no study has been conducted to investigate the potential roles of miRNAs in the joint injuries of KBD.

Recent studies found that changes in miRNAs expression profiles were associated with multiple complex human diseases, such as OA and osteoporosis^9, 10^. Because of the stability, non-invasiveness and sensitiveness of miRNAs detection, abnormally expressed miRNAs have the potential to be used as biomarkers for disease diagnosis^11^.

In this study, we compared the miRNAs expression profiles of KBD, OA, RA and healthy controls. We observed altered miRNAs expression profiles of KBD comparing to healthy controls. We also identified a set of miRNAs expressed differently in KBD vs. OA and KBD vs. RA. To the best of our knowledge, this study is the first miRNAs study of KBD. Our study results may help to understand the role of miRNAs in the development of KBD. Differently expressed miRNAs identified by this study also provide useful information for using miRNAs as the biomarkers for distinguishing KBD from OA and RA.

## Results

### Comparison of miRNA profiles among KBD, OA, RA patients and healthy controls

We identified 140 differently expressed miRNAs in KBD vs. Controls, including 118 up-regulated miRNAs and 22 down-regulated miRNA. We further compared the miRNAs expression profiles of KBD vs. OA and KBD vs. RA, respectively. In KBD vs. OA, we identified 123 differently expressed miRNAs, including 108 up-regulated miRNAs and 15 down-regulated miRNAs. In KBD vs. RA, we identified 34 differently expressed miRNAs, including 28 up-regulated miRNAs and 6 down-regulated miRNAs. To identify potential miRNAs biomarkers for distinguishing KBD from OA and RA, we further compared the differently expressed miRNAs in KBD vs. Controls, KBD vs. OA and KBD vs. RA. We detected 18 common miRNAs expressed differently in KBD vs. Controls, KBD vs. OA and KBD vs. RA (Table 1). Supplementary Figure S1 presents the miRNA heat map and hierarchical clustering images of KBD vs. Controls, KBD vs. OA and KBD vs. RA.

**Table 1:** List of 18 common miRNAs differently expressed in KBD vs. Control, KBD vs. OA and KBD vs. RA.

miRNAs	KBD vs. Control		KBD vs. OA		KBD vs. RA	
	ratio^a^	P value	ratio^a^	P value	ratio^a^	P value
hsa-miR-30d-3p	32.90	1.49E-03	30.79	9.39E-03	6.49	1.98 E-02
hsa-miR-522-3p	40.30	4.41 E-04	40.29	3.74 E-03	4.82	7.59 E-03
hsa-miR-4277	43.93	2.44 E-04	43.68	2.6 E-04	4.03	1.40 E-02
hsa-miR-548v	14.01	4.22 E-02	12.78	4.30 E-02	3.88	4.03 E-02
hsa-miR-3130-5p	6.35	6.33 E-03	8.63	6.56 E-03	3.82	1.25 E-02
hsa-miR-34b-3p	8.21	6.31 E-03	8.07	7.88 E-03	3.51	1.86 E-02
hsa-miR-764	5.74	6.77 E-05	9.11	1.53 E-04	3.46	6.77 E-03
hsa-miR-1267	6.39	2.38 E-03	10.45	1.06 E-02	3.37	8.23 E-03
hsa-miR-219-5p	5.02	4.04 E-03	5.31	3.89 E-03	2.91	1.43 E-02
hsa-miR-148a-5p	4.70	3.81 E-04	4.79	2.66 E-05	2.84	9.93 E-04
hsa-miR-4304	21.00	1.37 E-03	15.45	1.61 E-03	2.83	4.68 E-02
hsa-miRPlus-C1056	6.39	2.09 E-04	6.64	1.95 E-04	2.76	4.97 E-02
hsa-miRPlus-C1087	6.84	1.89 E-03	6.49	2.30 E-03	2.69	4.39 E-02
hsa-miR-2964a-5p	5.29	4.36 E-04	9.51	6.33 E-04	2.58	3.04 E-02
hsa-miR-30b-3p	11.96	2.24 E-04	8.29	4.76 E-04	2.41	1.77 E-02
hsa-miR-676-3p	3.04	2.84 E-03	4.54	2.24 E-03	2.07	2.70 E-02
hsa-miR-30e-3p	3.20	4.82 E-04	3.41	2.75 E-04	2.00	3.00 E-03
hsa-miR-720	0.45	1.20 E-03	0.36	1.53 E-02	0.51	2.47 E-02

^a^The differently expressed miRNAs passed volcano plot filtering and were identified at fold changes ≥2.0 and *P*-value ≤ 0.05.

### Gene ontology analysis of target genes of differently expressed miRNAs

To gain insight into the role of miRNAs in the joint injuries of KBD, the target genes of differently expressed miRNAs in KBD vs. Controls were predicted by miRror. We obtained a total of 125 miRNAs target genes (Supplementary Table S1), which were further imported into DAVID tool for gene ontology (GO) enrichment analysis. We identified 20 overrepresented GO terms with *P* value < 0.05, functionally mainly involved in Wnt receptor signaling pathway, hypoxia, vitamin B6 biosynthesis and metabolism (Table 2).

**Table 2:** Gene ontology enrichment analysis of target genes of differently expressed miRNAs in KBD vs. Control.

Gene category name	ID	P value
Plasma membrane part	GO:0044459	7.52E-04
Cytoplasmic vesicle	GO:0031410	1.06E-03
Vesicle	GO:0031982	1.52E-03
Cytoplasmic membrane-bounded vesicle	GO:0016023	3.45E-03
Membrane-bounded vesicle	GO:0031988	4.33E-03
Extrinsic to membrane	GO:0019898	5.44E-03
Wnt receptor signaling pathway	GO:0016055	1.19E-02
Response to hypoxia	GO:0001666	1.22E-02
Golgi apparatus	GO:0005794	1.24E-02
Golgi apparatus part	GO:0044431	1.35E-02
Response to oxygen levels	GO:0070482	1.45E-02
Vitamin biosynthetic process	GO:0009110	1.48E-02
RNA polymerase II transcription factor activity	GO:0003702	1.49E-02
Positive regulation of endocytosis	GO:0045807	1.58E-02
Vitamin B6 metabolic process	GO:0042816	1.98E-02
Vitamin B6 biosynthetic process	GO:0042819	1.98E-02
Cell junction	GO:0030054	2.16E-02
Golgi membrane	GO:0000139	3.53E-02
Procollagen-proline dioxygenase activity	GO:0019798	4.08E-02
Peptidyl-proline dioxygenase activity	GO:0031543	4.65E-02

### Integrating miRNA and mRNA expression profiles of KBD vs. Control

Message RNA (mRNA) expression profiling identified 97 differently expression genes, including 83 up-regulated genes and 14 down-regulated genes. Comparing the lists of predicted miRNA target genes and mRNA differently expressed genes detected 6 common genes for KBD, including LRRCC1 (mRNA expression ratio = 1.92), MS4A1 (mRNA expression ratio = 1.91), BUB3 (mRNA expression ratio = 1.61), CAPS2 (mRNA expression ratio = 1.54), C8orf33 (mRNA expression ratio = 1.50) and PDHA1 (mRNA expression ratio = 1.50).

## Discussion

Early treatments can greatly reduce the joint injuries of KBD. Current KBD diagnosis is mainly based on clinical manifestations and radiographic changes of KBD. The clinical manifestations of KBD are similar to that of OA and RA. Furthermore, there is not significant radiographic change in the early stage of KBD, making it very difficult to early diagnose KBD and distinguish KBD from OA and RA. In this study, we provides the first evidence for altered miRNAs expression profiles of KBD comparing to healthy controls, OA and RA.

Recent studies have demonstrated the implication of altered miRNAs expression profiles in the development of OA and RA^7, 12^. Abnormally expressed miRNAs provided novel useful information for the pathogenetic studies of OA and RA^8, 12^. This study also provides clues for understanding the role of miRNAs in the development of KBD. Previous genome-wide gene expression profiling of KBD articular cartilage suggested that hypoxia involved in the development of KBD^13^. Our GO enrichment analysis observed that the Response to Hypoxia GO (GO:0001666) was significantly overrepresented in the target genes of differently expressed miRNAs in KBD vs. Control, providing novel evidence for the role of hypoxia in the joint injuries of KBD.

Wnt signal pathway is a key regulator of cartilage development and homeostasis^14, 15^. Previous studies have demonstrated the importance of Wnt signal pathway in the joint injuries of OA^14, 15^. In this study, GO enrichment analysis found that the Wnt Receptor Signaling GO (GO:0016055) was overrepresented in the target genes of differently expressed miRNAs in KBD vs. Controls. This result suggests that Wnt signal pathway also involved in the joint lesions of KBD.

Another interesting finding of this study is the potential role of vitamin B6 deficiency in the pathogenesis of KBD. GO enrichment analysis of miRNAs target genes found that Vitamin B6 Metabolic Process GO (GO:0042816) and Vitamin B6 Biosynthetic Process GO (GO:0042819) were overrepresented in the target genes of differently expressed miRNAs of KBD vs. Control. No relevance between vitamin B6 deficiency and KBD has been reported by now. But previous studies found that low level of vitamin B6 was associated with RA^16, 17^. Vitamin B6 supplement was able to suppress pro-inflammatory factors (such as IL-6 and TNF-a) in RA patients^18^. It is interesting that increased serum levels of pro-inflammatory factors have been observed in KBD patients^19–21^. Given the significant impact of nutrition deficiency on the risk of KBD^22–24^, low vitamin B6 status may contribute to the joint injuries of KBD. It should be noted that, a relative small sample was used for miRNA expression profiling of KBD, OA, RA and healthy controls in this study. Further studies with large samples and cell experiments are needed to confirm our finding and clarify the potential mechanism of differently expressed miRNAs involved in the development of KBD.

In summary, we conducted the first miRNAs study of KBD. Our results demonstrate the altered miRNAs expression profiles of KBD comparing to healthy controls, RA and OA. The identified miRNAs have the potential to serve as biomarkers for KBD diagnosis and differential diagnosis.

## Materials and Methods

### Ethics statement

This study was designed according to the Declaration of Helsinki's ethical principles and was approved by the Institutional Review Board of Xi’an Jiaotong University. The methods were performed in accordance with the approved guidelines and regulations. Written Informed consent was obtained from all subjects.

### Patients

**Figure 1:**
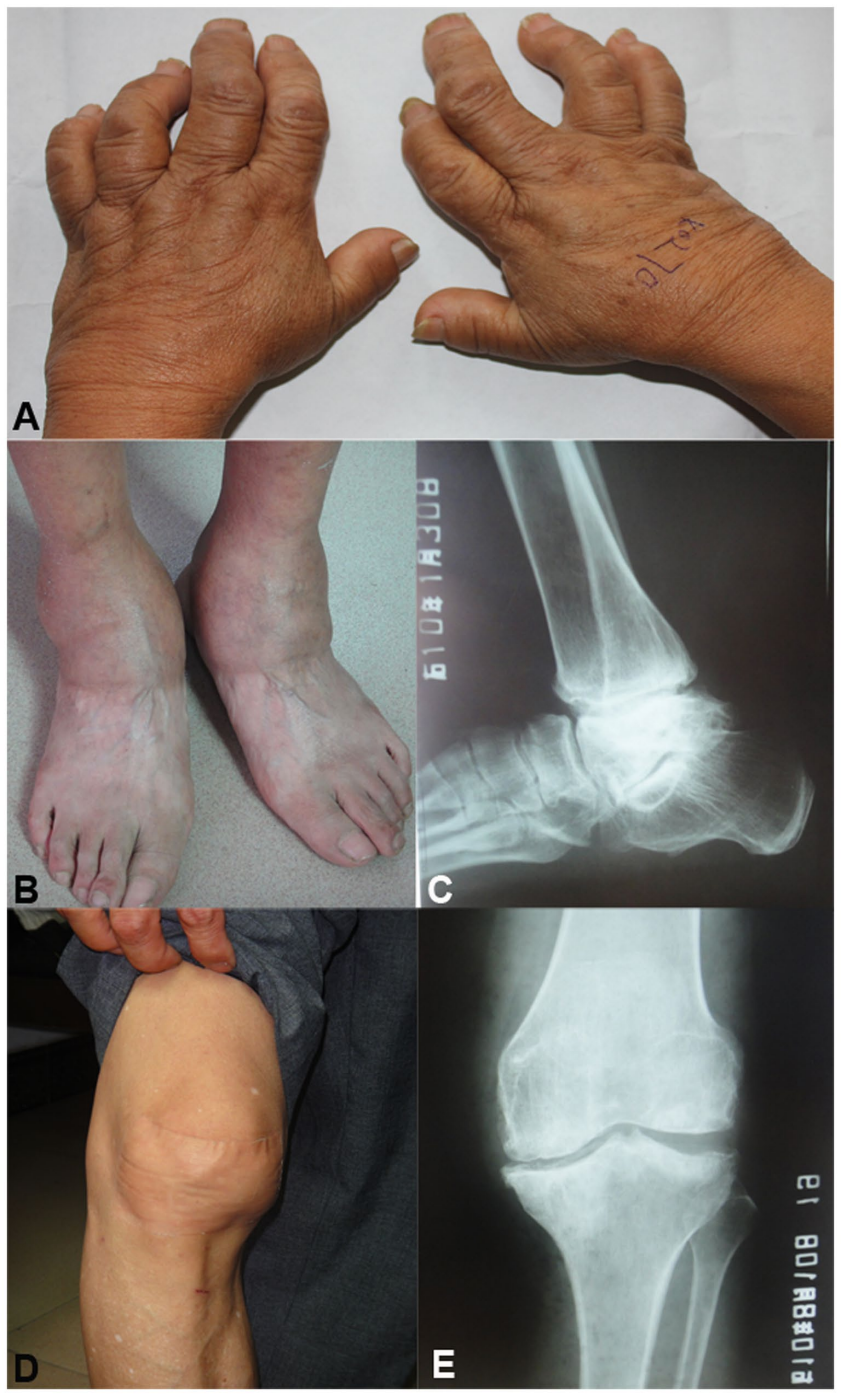
Characteristics of study samples with Kashin-Beck disease. (**A**) Images of hands; (**B**,**C**) image and radiographic image of ankle; (**D**,**E**) image and radiographic image of knee.

The KBD patients were collected from KBD-endemic area Yongshou county of Xi’an city of China (Fig. 1). The OA patients, RA patients and healthy controls came from non KBD-endemic areas of Xi’an city, and were collected at the first affiliated hospital of Xi’an Jiaotong University. Totally, 16 Kashin-Beck diseases (KBD) patients, 16 osteoarthritis (OA) patients, 16 rheumatoid arthritis (RA) patients and 16 healthy controls, were divided into three groups for miRNA profiling, with their ages and genders matched (Table 3). With regard to KBD patients, 10 grade II patients and 6 grade III patients are included. All study subjects were Chinese Han and genetic unrelated. Based on a history of living in a KBD prevalent area, clinical manifestations (typically short fingers, enlargement of finger joints, flexion of the distal finger and deformed fingers in hands) and radiography of right hand (typically irregular sclerosis and reappearance of calcification zone in the metaphysis of metacarpus and phalange), KBD was diagnosed according to the national diagnosis criteria of KBD in China (Diagnostic code: WS/T 207-2010). OA and RA were diagnosed according to the diagnosis criteria of the American College of Rheumatology (ACR). The subjects with other bone and joint diseases, rheumatic diseases or receiving drug therapies (including NSAIDs, steroids, methotrexate and so on) in the six months before sample recruiting time were excluded from this study.

**Table 3:** Basic characteristics of study subjects for miRNA expression profile study.

Group	KBD		OA		RA		Healthy control	
	N	Age (range)	N	Age (range)	N	Age (range)	N	Age (range)
1	6	48 (40–54)	6	51 (46–55)	6	48 (42–57)	6	49 (39–53)
2	5	60 (54–63)	5	58 (54–61)	5	55 (50–58)	5	56 (50–62)
3	5	71 (68–72)	5	67 (63–71)	5	67 (62–71)	5	66 (60–71)
Total	16	59 (40–72)	16	58 (46–71)	16	56 (42–71)	16	56 (39–71)

### Total RNA isolation

5 ml blood specimens were collected from each study subject into sterile EDTA tubes and transferred into sterile RNase-free centrifuge tubes in a super-clean bench used to extract total RNA immediately after received the samples. Total RNA was isolated and enriched using Invitrogen^TM^ TRIzol (Thermo Fisher Scientific Inc, Wilmington, DE, USA) and miRNeasy mini kit (QIAGEN, Hilden, Germany) following manufacturer recommended protocol. RNA was isolated from the whole blood by centrifuging at 12,000 g, 4 °C. Main procedures are as shown below. Add 0.2 ml of whole blood to 0.75 ml of TRI Reagent BD supplemented with 20 ul of 5 N acetic acid per 0.2 ml of whole blood; close the tube and shake the resulting mixture well by hand; store the lysed samples for 5 minutes at room temperature; supplement the lysate with 0.2 ml of chloroform per 0.75 ml of TRI Reagent BD, cover the samples tightly and shake vigorously for 15 seconds; store the resulting mixture at room temperature for 2–5 minutes and centrifuge at 12,000 g for 15 minutes at 4 °C; transfer the aqueous phase (RNA remaining) to a fresh tube, and save the interphase and organic phase at 4 °C for subsequent isolation of DNA and proteins; precipitate RNA from the aqueous phase by mixing with isopropanol, store samples at room temperature for 5–10 min and centrifuge at 12,000 g for 8 minutes at 4 °C; remove the supernatant and mix the RNA pellet in 75% ethanol by vortexing; centrifuge the RNA suspension at 12,000 g for 5 minutes at 4 °C; remove the ethanol wash and briefly air-dry the RNA pellet for 5 min. For enrichment of miRNAs, dissolve RNA in nuclease-free water and add ethanol to provide appropriate binding conditions. The sample is then applied to the RNeasy Mini spin column, where the total RNA binds to the membrane and phenol and other contaminants are efficiently washed away. High-quality RNA is then eluted in RNase-free water. The quality of isolated total RNA was evaluated by gel electrophoresis. The concentrations of isolated total RNA were determined by NanoDrop spectrophotometer ND-1000 (Thermo Fisher Scientific Inc, Wilmington, DE, USA). For each pooling specimen, the same amount of RNA of each subject was mixed together according to determined concentrations.

### miRNAs microarray hybridization

miRNAs expression profiling was performed using single-channel miRCURY LNA^TM^ miRNAs Array v16.0 (Exiqon, Inc., Woburn, MA, USA). miRCURY LNA^TM^ miRNAs Array v16.0 contains 1,891 capture probes, covering all human miRNAs annotated in the miRBase (release 16, http://mirbase.org/) and 66 new miRPlus^TM^ human miRNAs. The isolated total RNA of each subject was labeled using miRCURY^TM^ Power labeling kit (Exiqon, Inc., Woburn, MA, USA), and hybridized to miRCURY LNA^TM^ miRNAs Array v16.0 following Exiqon recommended standard protocol. Hybridization image scanning was performed by Axon GenePix 4000B microarray scanner (Molecular Devices, LLC., Sunnyvale, CA, USA). Scanned images were imported into GenePix Pro 6.0 software (Molecular Devices, LLC., Sunnyvale, CA, USA) for data extraction, median normalization and quality control. Our microarray data was MIAME compliant and has been deposited in the MIAME compliant database ArrayExpress (Accession number: E-MTAB-3514).

### miRNAs microarray analysis and target gene prediction

Raw signal intensities were normalized using Median normalization approach and chosen for differentially expressed miRNAs screening. The miRNAs with intensities >50 in all samples were chosen for calculating Median normalization factor. Volcano Plot filtering was performed to identify differently expressed miRNAs with fold changes ≥2.0 and *P*-value ≤ 0.05. miRNAs target genes were predicted by miRror suite (http://www.proto.cs.huji.ac.il/mirror/index.php)^25^. miRror provides a unified miRNAs target gene prediction platform covering most of current popular miRNAs target predicting tools, such as TargetScan^26^, RNA22^27^ and miRDB^28^. To ensure the accuracy of miRNAs target prediction, the target genes (miRror *P* value < 0.01) predicted by at least two predicting tools of miRror were included in this study.

### GO enrichment analysis

GO enrichment analysis of target genes of differently expressed miRNAs in KBD vs. Control was performed by functional annotation bioinformatics tool DAVID (version 6.7, http://david.abcc.ncifcrf.gov/)^29, 30^.

### mRNA expression profiling of KBD vs. Controls

Our previous mRNA expression profile dataset of 20 KBD patients and 15 healthy controls were used here. Briefly, the total RNA was isolated from peripheral blood using Trizol reagent (Life Technologies Inc., Carlsbad, California, USA), transcribed into aRNA and labeled with CyDye using Amino Allyl MessageAmp aRNA Kit (Applied Biosystems, Austin, TX, USA). Agilent Human 1A 22 k Whole Genome microarray (G4110B) that contains 22,575 oligonucleotides probes representing 21,073 human genes, was applied for microarray hybridization according to the manufacturer's protocol. Hybridization signals were recorded by Gene-Pix 4000B (Axon Instruments Inc., Foster City, California, USA), and analyzed by Feature Extraction 9.3 (Agilent Technologies) and Spotfire 8.0 (Spotfire Inc., Cambridge, MA, USA) software. The fluorescent spots that failed to pass the quality control procedure were excluded for further analysis. Expression ratio was calculated to evaluate the significance of expression difference between KBD and control samples. Our microarray data are MIAME compliant and have been deposited in GEO database (Accession number: GSE32127). Detailed description of sample characteristics, experiment design and statistical analysis can be found in our previous study^31^.

## Electronic supplementary material

Heat Map and Hierarchical Clustering of differentially expressed miRNAs of KBD vs. Control, KBD vs. OA, KBD vs. RA.

List of target genes of miRNAs differently expressed in KBD vs. Control
