# Supplementary material for: Comparison of microRNA expression profiles of Kashin-Beck disease, osteoarthritis and rheumatoid arthritis
Source: Sci Rep. 2017 Apr 3;7:540. doi: 10.1038/s41598-017-00522-z (PMC5428653; doi:10.1038/s41598-017-00522-z)
Supplement: Supplementary file 1 — Heat Map and Hierarchical Clustering of differentially expressed miRNAs of KBD vs. Control, KBD vs. OA, KBD vs. RA. [file 41598_2017_522_MOESM1_ESM.pdf]

Comparison of microRNA expression profiles of Kashin-Beck disease, osteoarthritis and rheumatoid arthritis

Wenhong Wu[#1], Awen He[#1], Yan Wen[1], Xiao Xiao[1], Jingcan Hao[1], Feng Zhang[1]*, Xiong Guo[1]*

Figure S1. Heat Map and Hierarchical Clustering of differentially expressed miRNAs of KBD vs. Control, KBD vs. OA, KBD vs. RA.

The heat map diagram shows the result of the two-way hierarchical clustering of miRNAs and samples. Each row represents a miRNA and each column represents a sample. The miRNA clustering tree is shown on the left, and the sample clustering tree appears at the top. The color scale shown at the top illustrates the relative expression level of a miRNA in the certain slide: red color represents a high relative expression level ; green color represents a low relative expression levels.

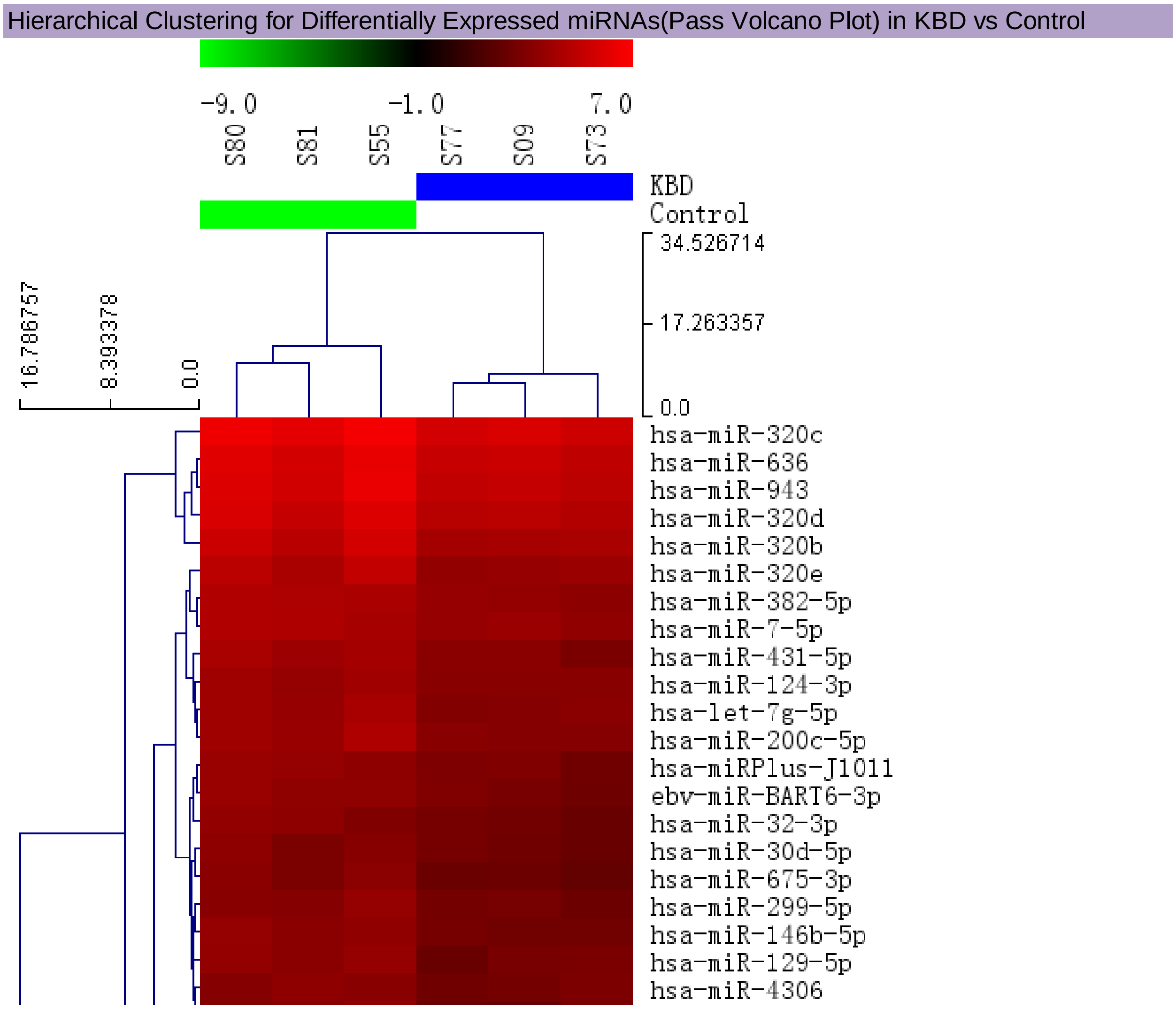

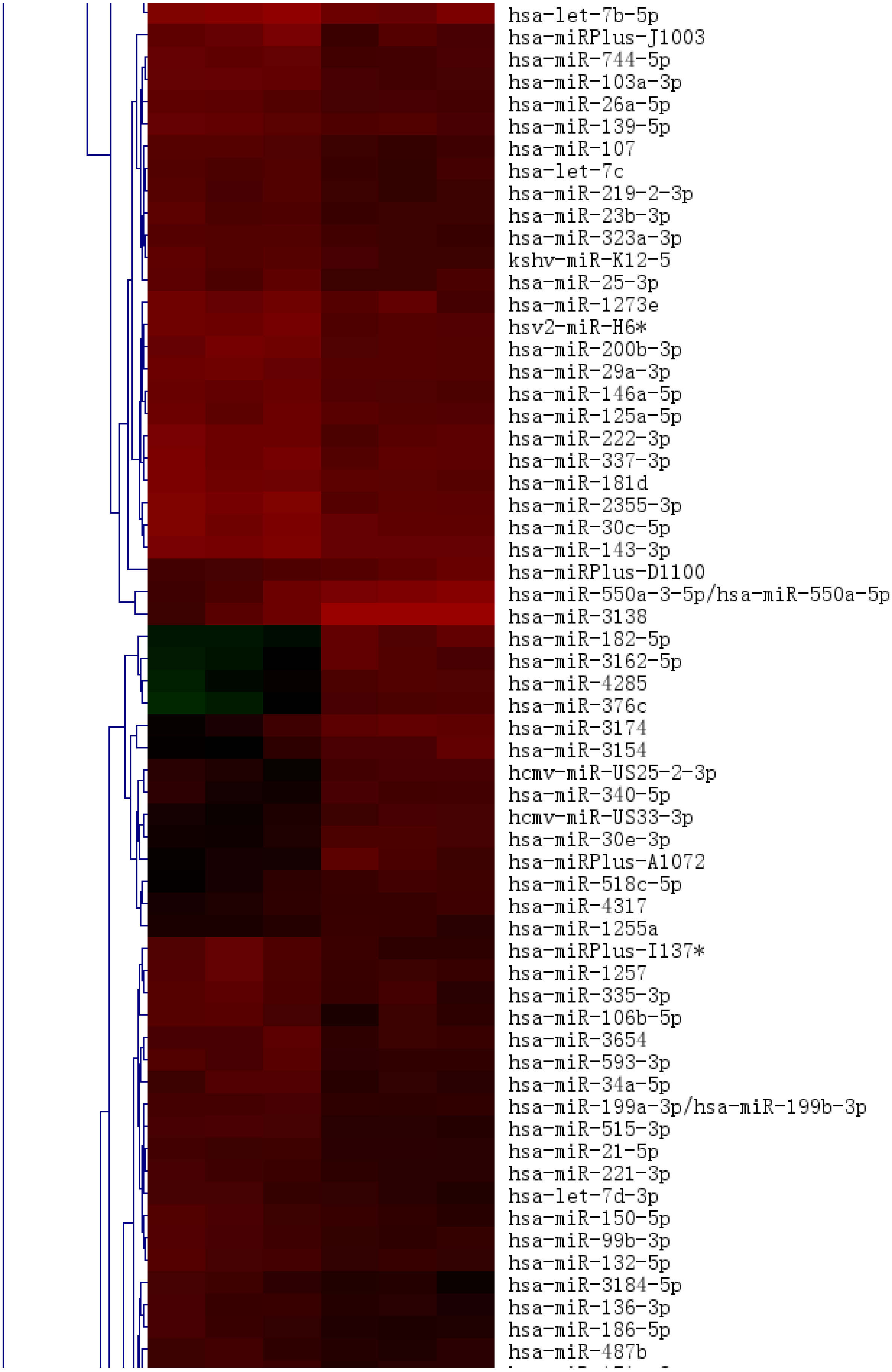

hsa-let-7b-5p
hsa-miRPlus-J1003
hsa-miR-744-5p
hsa-miR-103a-3p
hsa-miR-26a-5p
hsa-miR-139-5p
hsa-miR-107
hsa-let-7c
hsa-miR-219-2-3p
hsa-miR-23b-3p
hsa-miR-323a-3p
kshv-miR-K12-5
hsa-miR-25-3p
hsa-miR-1273e
hsv2-miR-H6*
hsa-miR-200b-3p
hsa-miR-29a-3p
hsa-miR-146a-5p
hsa-miR-125a-5p
hsa-miR-222-3p
hsa-miR-337-3p
hsa-miR-181d
hsa-miR-2355-3p
hsa-miR-30c-5p
hsa-miR-143-3p
hsa-miRPlus-D1100
hsa-miR-550a-3-5p/hsa-miR-550a-5p
hsa-miR-3138
hsa-miR-182-5p
hsa-miR-3162-5p
hsa-miR-4285
hsa-miR-376c
hsa-miR-3174
hsa-miR-3154
hcmv-miR-US25-2-3p
hsa-miR-340-5p
hcmv-miR-US33-3p
hsa-miR-30e-3p
hsa-miRPlus-A1072
hsa-miR-518c-5p
hsa-miR-4317
hsa-miR-1255a
hsa-miRPlus-I137*
hsa-miR-1257
hsa-miR-335-3p
hsa-miR-106b-5p
hsa-miR-3654
hsa-miR-593-3p
hsa-miR-34a-5p
hsa-miR-199a-3p/hsa-miR-199b-3p
hsa-miR-515-3p
hsa-miR-21-5p
hsa-miR-221-3p
hsa-let-7d-3p
hsa-miR-150-5p
hsa-miR-99b-3p
hsa-miR-132-5p
hsa-miR-3184-5p
hsa-miR-136-3p
hsa-miR-186-5p
hsa-miR-487b

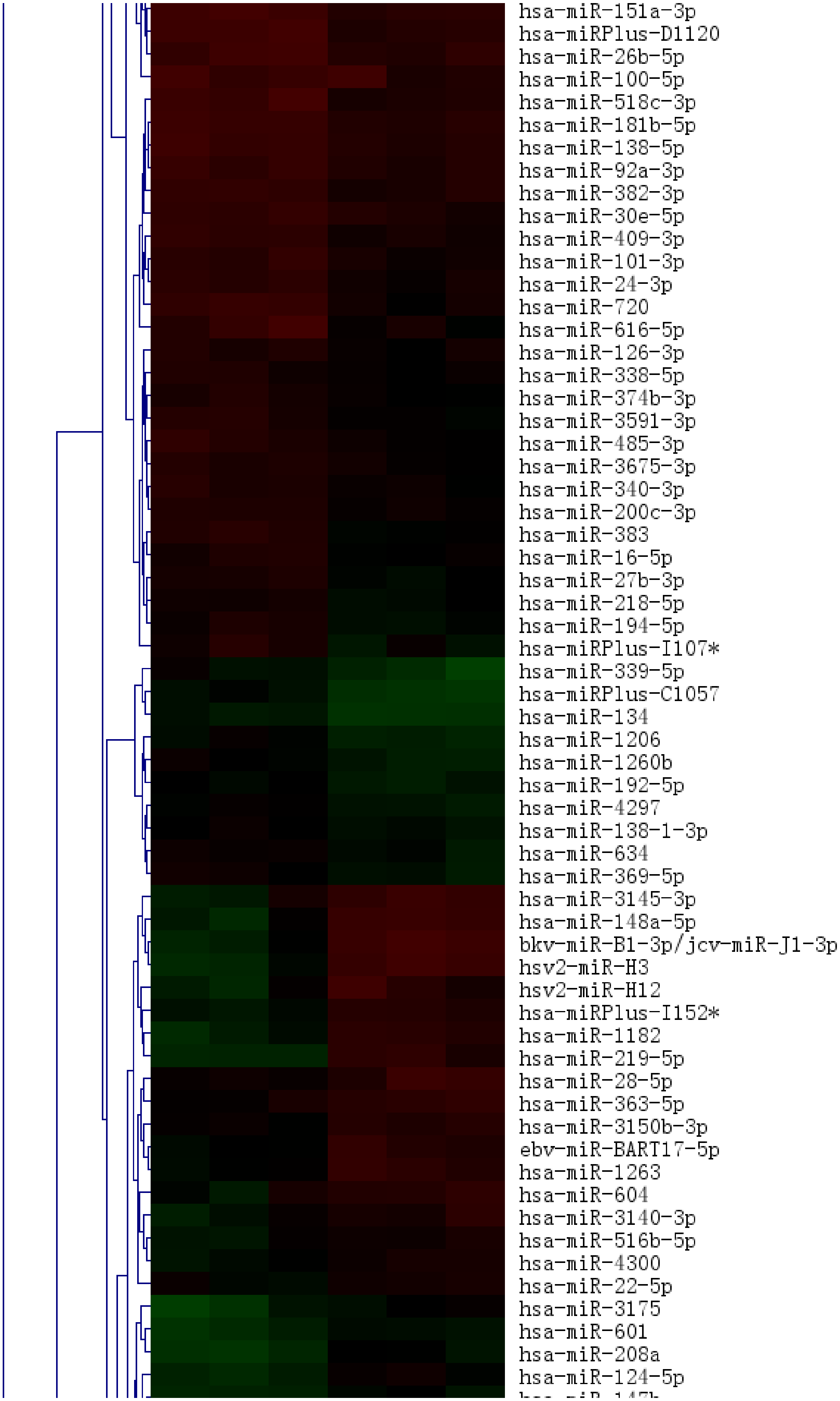
hsa-miR-151a-3p
hsa-miRPlus-D1120
hsa-miR-26b-5p
hsa-miR-100-5p
hsa-miR-518c-3p
hsa-miR-181b-5p
hsa-miR-138-5p
hsa-miR-92a-3p
hsa-miR-382-3p
hsa-miR-30e-5p
hsa-miR-409-3p
hsa-miR-101-3p
hsa-miR-24-3p
hsa-miR-720
hsa-miR-616-5p
hsa-miR-126-3p
hsa-miR-338-5p
hsa-miR-374b-3p
hsa-miR-3591-3p
hsa-miR-485-3p
hsa-miR-3675-3p
hsa-miR-340-3p
hsa-miR-200c-3p
hsa-miR-383
hsa-miR-16-5p
hsa-miR-27b-3p
hsa-miR-218-5p
hsa-miR-194-5p
hsa-miRPlus-I107*
hsa-miR-339-5p
hsa-miRPlus-C1057
hsa-miR-134
hsa-miR-1206
hsa-miR-1260b
hsa-miR-192-5p
hsa-miR-4297
hsa-miR-138-1-3p
hsa-miR-634
hsa-miR-369-5p
hsa-miR-3145-3p
hsa-miR-148a-5p
bkv-miR-B1-3p/jcv-miR-J1-3p
hsv2-miR-H3
hsv2-miR-H12
hsa-miRPlus-I152*
hsa-miR-1182
hsa-miR-219-5p
hsa-miR-28-5p
hsa-miR-363-5p
hsa-miR-3150b-3p
ebv-miR-BART17-5p
hsa-miR-1263
hsa-miR-604
hsa-miR-3140-3p
hsa-miR-516b-5p
hsa-miR-4300
hsa-miR-22-5p
hsa-miR-3175
hsa-miR-601
hsa-miR-208a
hsa-miR-124-5p

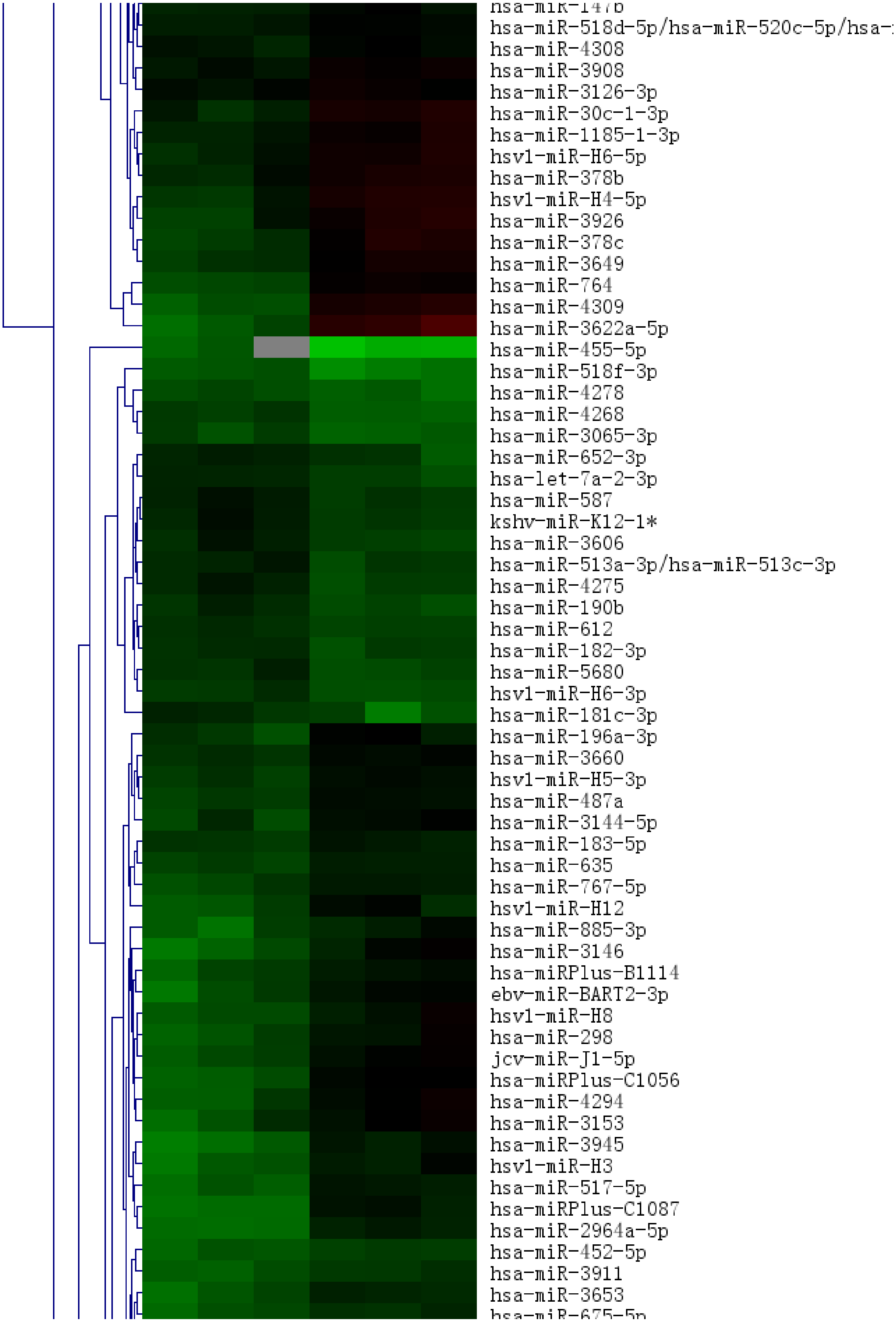
hsa-miR-518d-5p/hsa-miR-520c-5p/hsa-
hsa-miR-4308
hsa-miR-3908
hsa-miR-3126-3p
hsa-miR-30c-1-3p
hsa-miR-1185-1-3p
hsv1-miR-H6-5p
hsa-miR-378b
hsv1-miR-H4-5p
hsa-miR-3926
hsa-miR-378c
hsa-miR-3649
hsa-miR-764
hsa-miR-4309
hsa-miR-3622a-5p
hsa-miR-455-5p
hsa-miR-518f-3p
hsa-miR-4278
hsa-miR-4268
hsa-miR-3065-3p
hsa-miR-652-3p
hsa-let-7a-2-3p
hsa-miR-587
kshv-miR-K12-1*
hsa-miR-3606
hsa-miR-513a-3p/hsa-miR-513c-3p
hsa-miR-4275
hsa-miR-190b
hsa-miR-612
hsa-miR-182-3p
hsa-miR-5680
hsv1-miR-H6-3p
hsa-miR-181c-3p
hsa-miR-196a-3p
hsa-miR-3660
hsv1-miR-H5-3p
hsa-miR-487a
hsa-miR-3144-5p
hsa-miR-183-5p
hsa-miR-635
hsa-miR-767-5p
hsv1-miR-H12
hsa-miR-885-3p
hsa-miR-3146
hsa-miRPlus-B1114
ebv-miR-BART2-3p
hsv1-miR-H8
hsa-miR-298
jcv-miR-J1-5p
hsa-miRPlus-C1056
hsa-miR-4294
hsa-miR-3153
hsa-miR-3945
hsv1-miR-H3
hsa-miR-517-5p
hsa-miRPlus-C1087
hsa-miR-2964a-5p
hsa-miR-452-5p
hsa-miR-3911
hsa-miR-3653

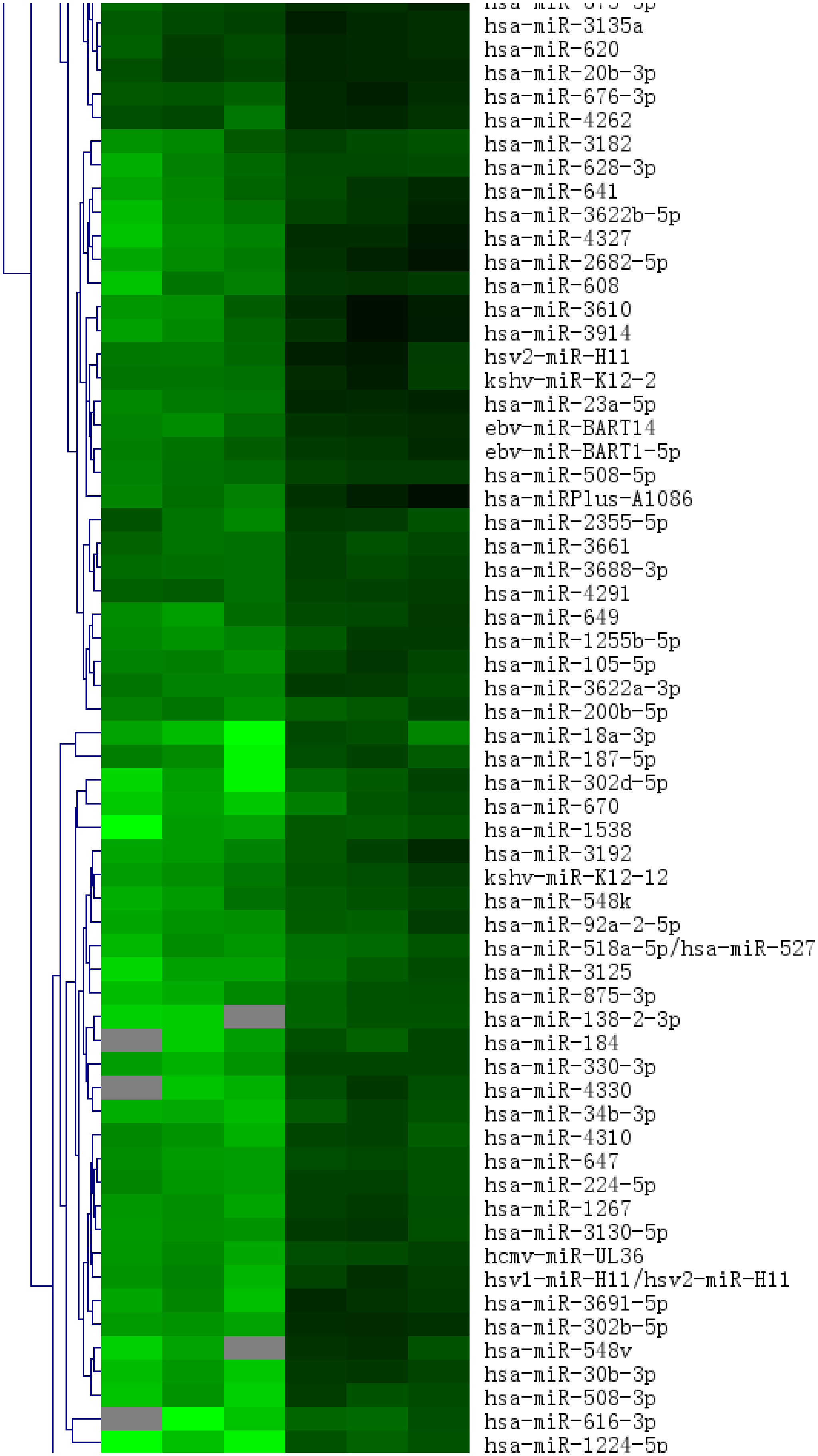
hsa-miR-3135a
hsa-miR-620
hsa-miR-20b-3p
hsa-miR-676-3p
hsa-miR-4262
hsa-miR-3182
hsa-miR-628-3p
hsa-miR-641
hsa-miR-3622b-5p
hsa-miR-4327
hsa-miR-2682-5p
hsa-miR-608
hsa-miR-3610
hsa-miR-3914
hsv2-miR-H11
kshv-miR-K12-2
hsa-miR-23a-5p
ebv-miR-BART14
ebv-miR-BART1-5p
hsa-miR-508-5p
hsa-miRPlus-A1086
hsa-miR-2355-5p
hsa-miR-3661
hsa-miR-3688-3p
hsa-miR-4291
hsa-miR-649
hsa-miR-1255b-5p
hsa-miR-105-5p
hsa-miR-3622a-3p
hsa-miR-200b-5p
hsa-miR-18a-3p
hsa-miR-187-5p
hsa-miR-302d-5p
hsa-miR-670
hsa-miR-1538
hsa-miR-3192
kshv-miR-K12-12
hsa-miR-548k
hsa-miR-92a-2-5p
hsa-miR-518a-5p/hsa-miR-527
hsa-miR-3125
hsa-miR-875-3p
hsa-miR-138-2-3p
hsa-miR-184
hsa-miR-330-3p
hsa-miR-4330
hsa-miR-34b-3p
hsa-miR-4310
hsa-miR-647
hsa-miR-224-5p
hsa-miR-1267
hsa-miR-3130-5p
hcmv-miR-UL36
hsv1-miR-H11/hsv2-miR-H11
hsa-miR-3691-5p
hsa-miR-302b-5p
hsa-miR-548v
hsa-miR-30b-3p
hsa-miR-508-3p
hsa-miR-616-3p
hsa-miR-1224-5p

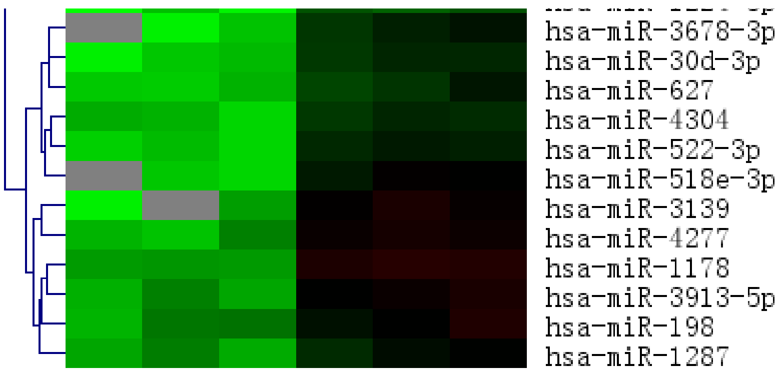

Hierarchical Clustering for Differentially Expressed miRNAs(Pass Volcano Plot) in KBD vs OA

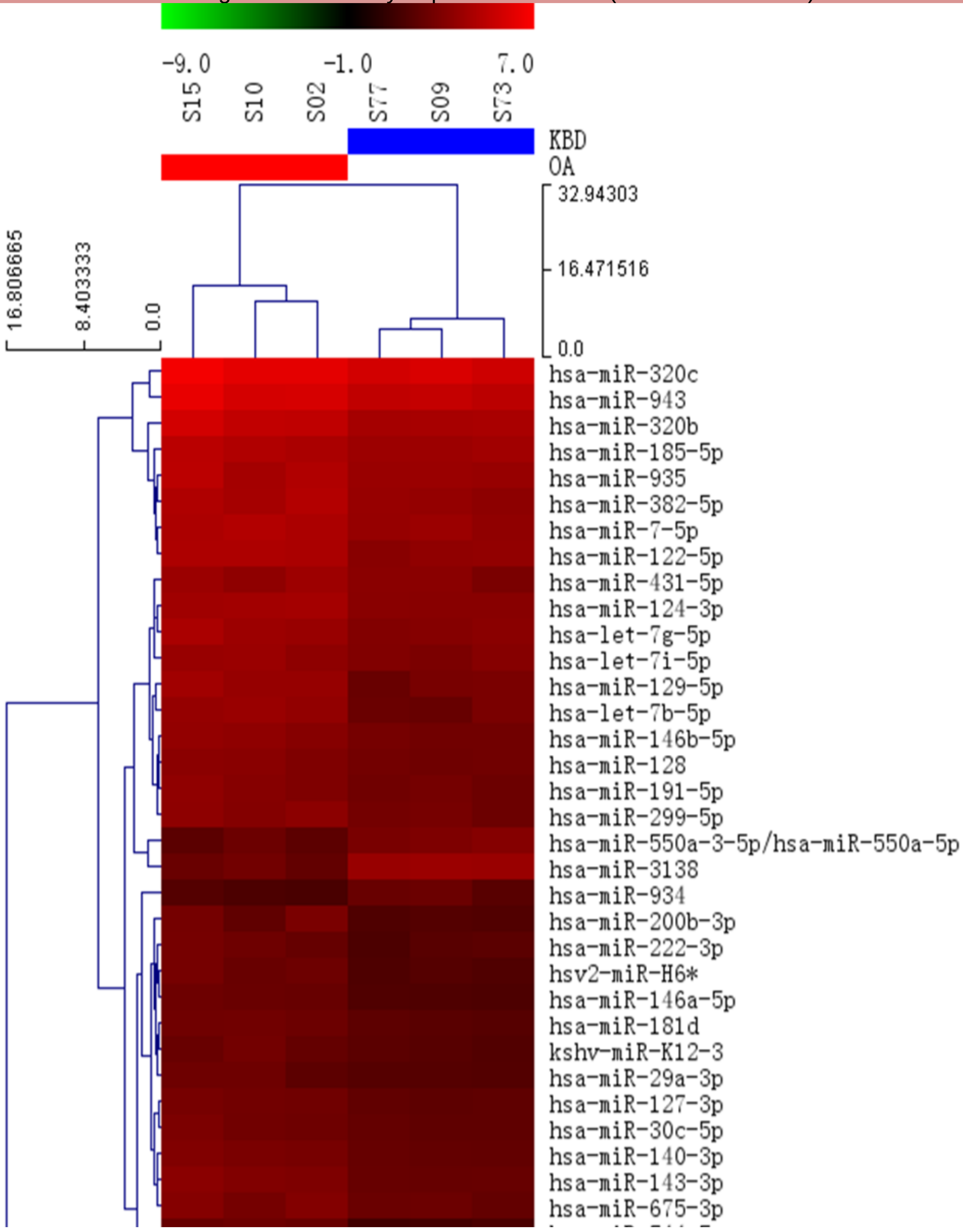

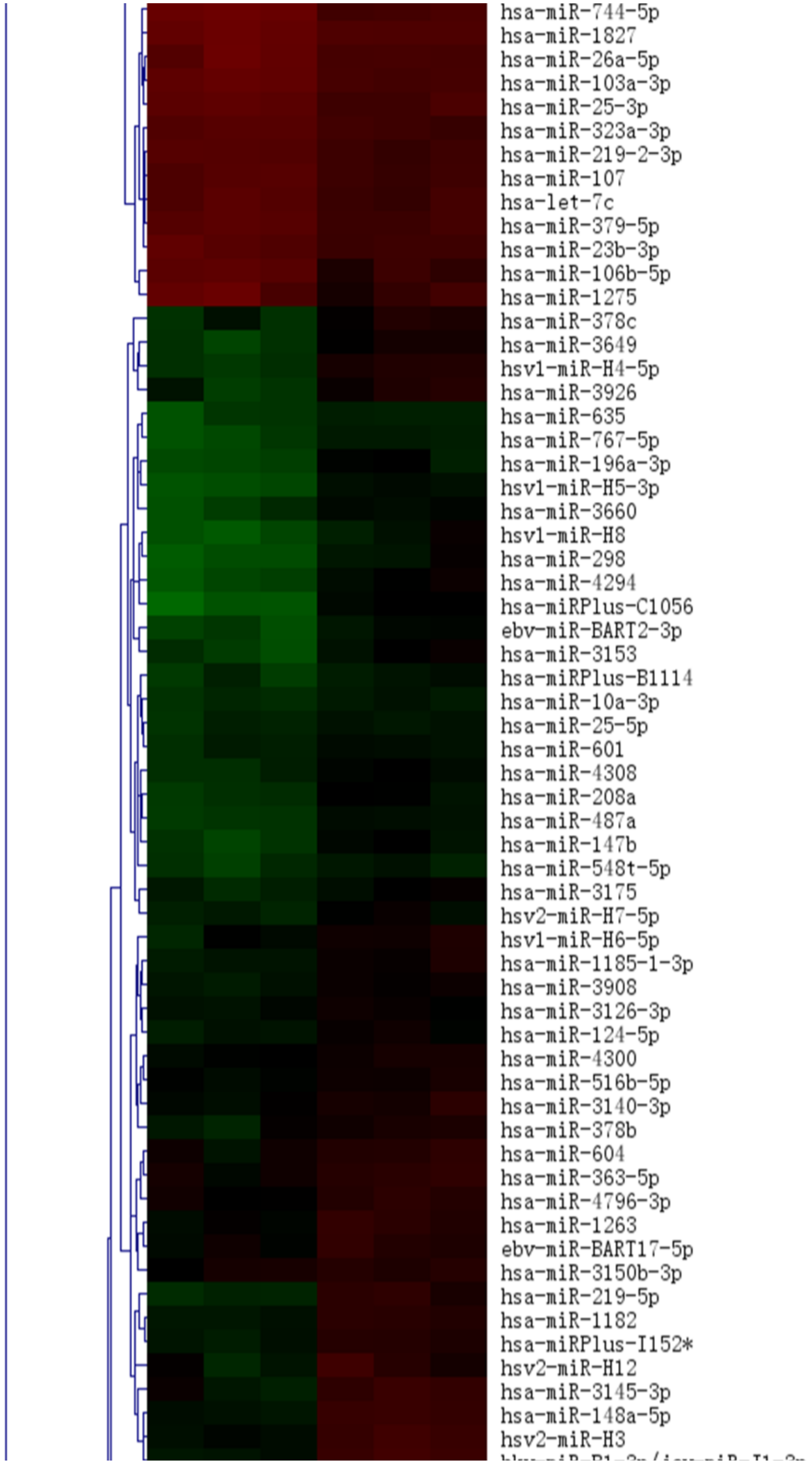
hsa-miR-744-5p
hsa-miR-1827
hsa-miR-26a-5p
hsa-miR-103a-3p
hsa-miR-25-3p
hsa-miR-323a-3p
hsa-miR-219-2-3p
hsa-miR-107
hsa-let-7c
hsa-miR-379-5p
hsa-miR-23b-3p
hsa-miR-106b-5p
hsa-miR-1275
hsa-miR-378c
hsa-miR-3649
hsv1-miR-H4-5p
hsa-miR-3926
hsa-miR-635
hsa-miR-767-5p
hsa-miR-196a-3p
hsv1-miR-H5-3p
hsa-miR-3660
hsv1-miR-H8
hsa-miR-298
hsa-miR-4294
hsa-miRPlus-C1056
ebv-miR-BART2-3p
hsa-miR-3153
hsa-miRPlus-B1114
hsa-miR-10a-3p
hsa-miR-25-5p
hsa-miR-601
hsa-miR-4308
hsa-miR-208a
hsa-miR-487a
hsa-miR-147b
hsa-miR-548t-5p
hsa-miR-3175
hsv2-miR-H7-5p
hsv1-miR-H6-5p
hsa-miR-1185-1-3p
hsa-miR-3908
hsa-miR-3126-3p
hsa-miR-124-5p
hsa-miR-4300
hsa-miR-516b-5p
hsa-miR-3140-3p
hsa-miR-378b
hsa-miR-604
hsa-miR-363-5p
hsa-miR-4796-3p
hsa-miR-1263
ebv-miR-BART17-5p
hsa-miR-3150b-3p
hsa-miR-219-5p
hsa-miR-1182
hsa-miRPlus-I152*
hsv2-miR-H12
hsa-miR-3145-3p
hsa-miR-148a-5p
hsv2-miR-H3

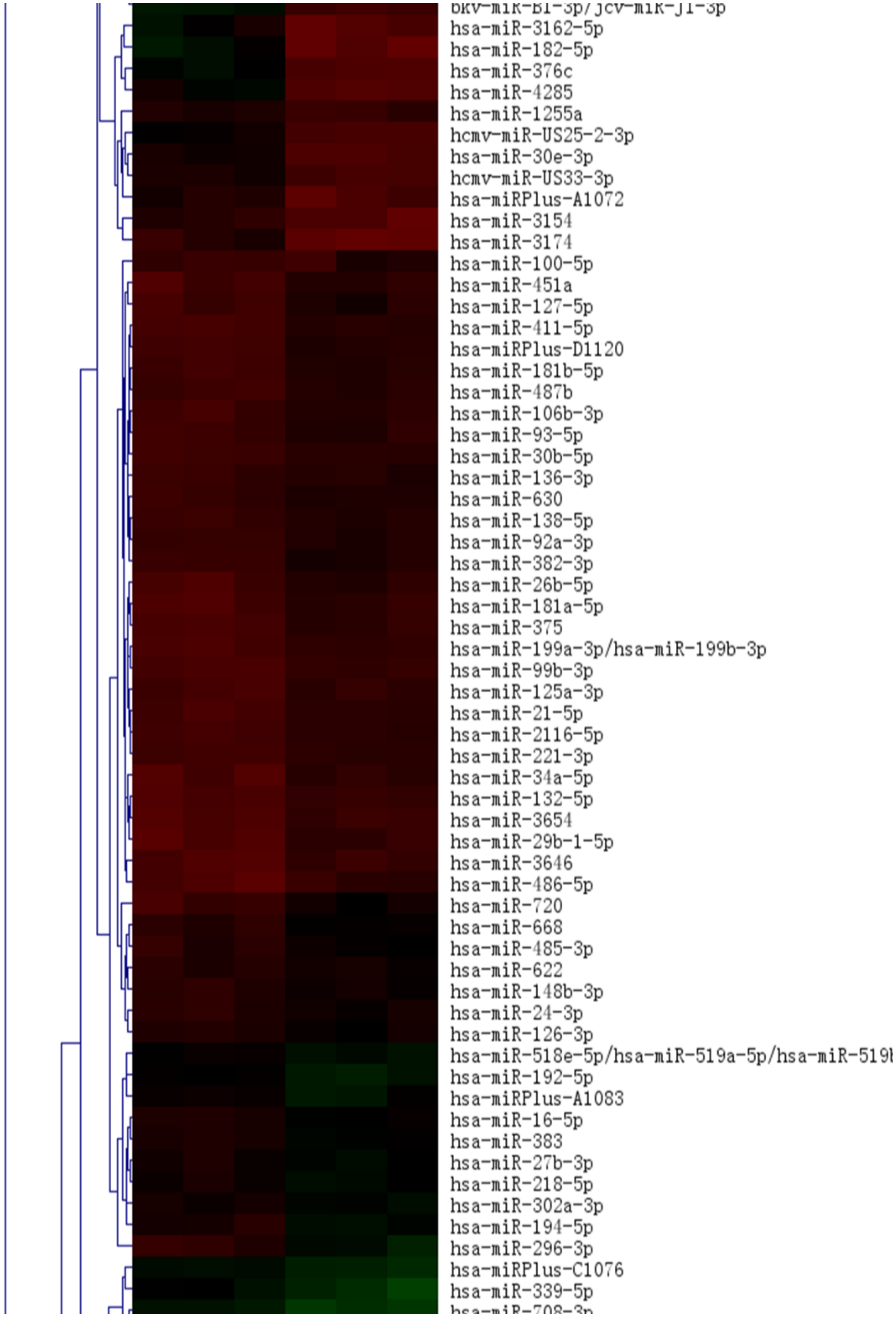
bkv-miR-B1-3p/jcv-miR-J1-3p
hsa-miR-3162-5p
hsa-miR-182-5p
hsa-miR-376c
hsa-miR-4285
hsa-miR-1255a
hcmv-miR-US25-2-3p
hsa-miR-30e-3p
hcmv-miR-US33-3p
hsa-miRPlus-A1072
hsa-miR-3154
hsa-miR-3174
hsa-miR-100-5p
hsa-miR-451a
hsa-miR-127-5p
hsa-miR-411-5p
hsa-miRPlus-D1120
hsa-miR-181b-5p
hsa-miR-487b
hsa-miR-106b-3p
hsa-miR-93-5p
hsa-miR-30b-5p
hsa-miR-136-3p
hsa-miR-630
hsa-miR-138-5p
hsa-miR-92a-3p
hsa-miR-382-3p
hsa-miR-26b-5p
hsa-miR-181a-5p
hsa-miR-375
hsa-miR-199a-3p/hsa-miR-199b-3p
hsa-miR-99b-3p
hsa-miR-125a-3p
hsa-miR-21-5p
hsa-miR-2116-5p
hsa-miR-221-3p
hsa-miR-34a-5p
hsa-miR-132-5p
hsa-miR-3654
hsa-miR-29b-1-5p
hsa-miR-3646
hsa-miR-486-5p
hsa-miR-720
hsa-miR-668
hsa-miR-485-3p
hsa-miR-622
hsa-miR-148b-3p
hsa-miR-24-3p
hsa-miR-126-3p
hsa-miR-518e-5p/hsa-miR-519a-5p/hsa-miR-519
hsa-miR-192-5p
hsa-miRPlus-A1083
hsa-miR-16-5p
hsa-miR-383
hsa-miR-27b-3p
hsa-miR-218-5p
hsa-miR-302a-3p
hsa-miR-194-5p
hsa-miR-296-3p
hsa-miRPlus-C1076
hsa-miR-339-5p
hsa-miR-708-3p

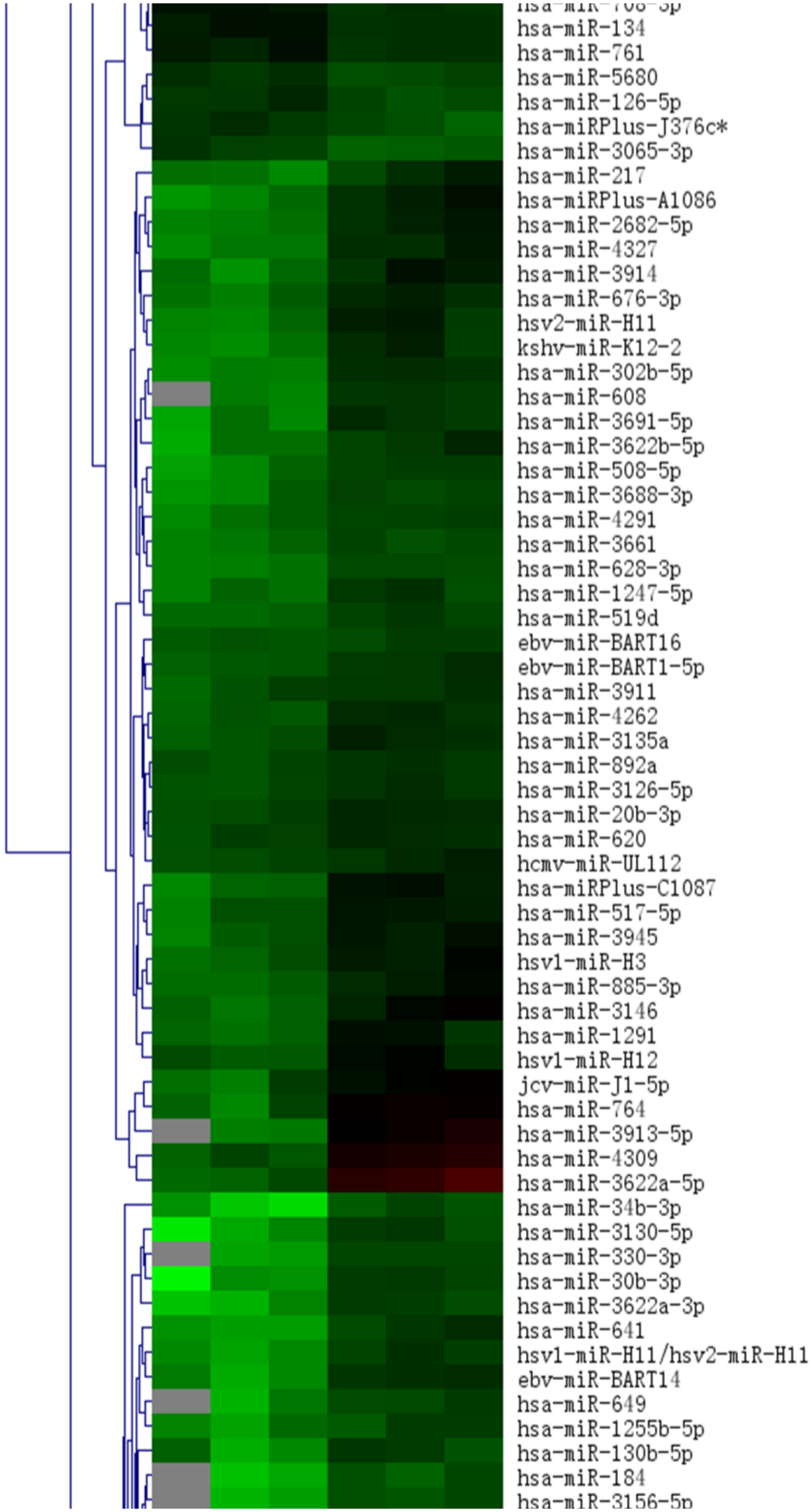
hsa-miR-134
hsa-miR-761
hsa-miR-5680
hsa-miR-126-5p
hsa-miRPlus-J376c*
hsa-miR-3065-3p
hsa-miR-217
hsa-miRPlus-A1086
hsa-miR-2682-5p
hsa-miR-4327
hsa-miR-3914
hsa-miR-676-3p
hsv2-miR-H11
kshv-miR-K12-2
hsa-miR-302b-5p
hsa-miR-608
hsa-miR-3691-5p
hsa-miR-3622b-5p
hsa-miR-508-5p
hsa-miR-3688-3p
hsa-miR-4291
hsa-miR-3661
hsa-miR-628-3p
hsa-miR-1247-5p
hsa-miR-519d
ebv-miR-BART16
ebv-miR-BART1-5p
hsa-miR-3911
hsa-miR-4262
hsa-miR-3135a
hsa-miR-892a
hsa-miR-3126-5p
hsa-miR-20b-3p
hsa-miR-620
hcmv-miR-UL112
hsa-miRPlus-C1087
hsa-miR-517-5p
hsa-miR-3945
hsv1-miR-H3
hsa-miR-885-3p
hsa-miR-3146
hsa-miR-1291
hsv1-miR-H12
jcv-miR-J1-5p
hsa-miR-764
hsa-miR-3913-5p
hsa-miR-4309
hsa-miR-3622a-5p
hsa-miR-34b-3p
hsa-miR-3130-5p
hsa-miR-330-3p
hsa-miR-30b-3p
hsa-miR-3622a-3p
hsa-miR-641
hsv1-miR-H11/hsv2-miR-H11
ebv-miR-BART14
hsa-miR-649
hsa-miR-1255b-5p
hsa-miR-130b-5p
hsa-miR-184
hsa-miR-3156-5p

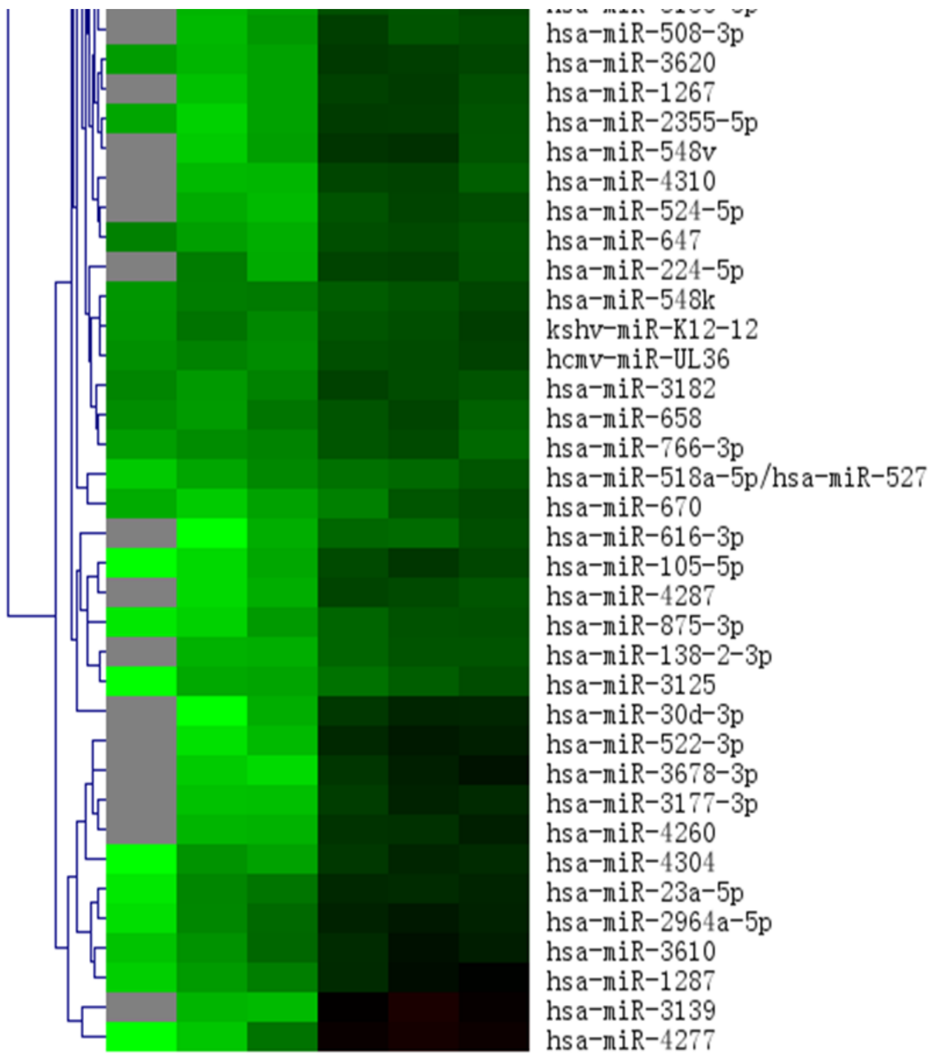

Hierarchical Clustering for Differentially Expressed miRNAs(Pass Volcano Plot) in KBD vs RA

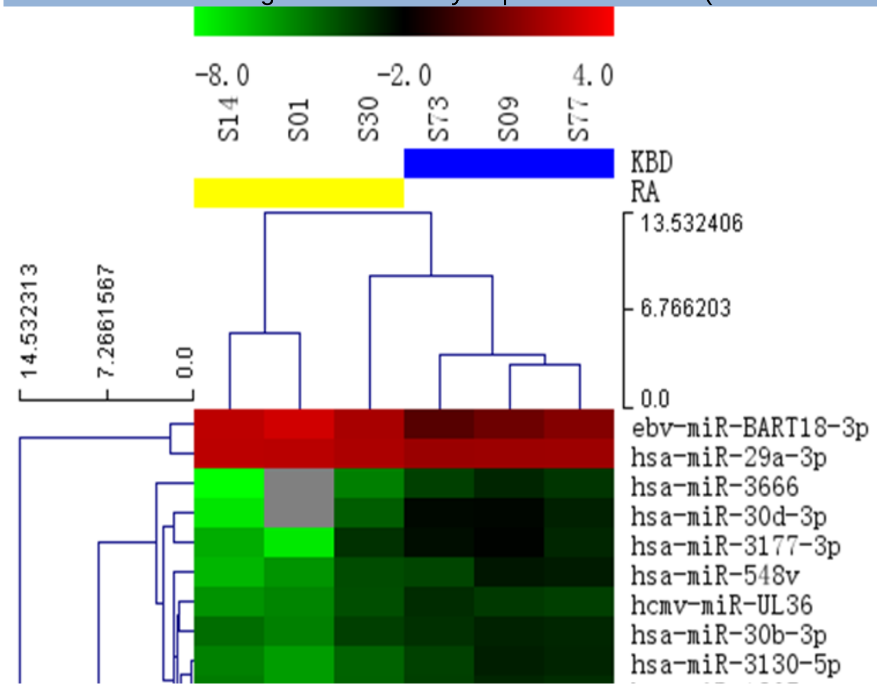

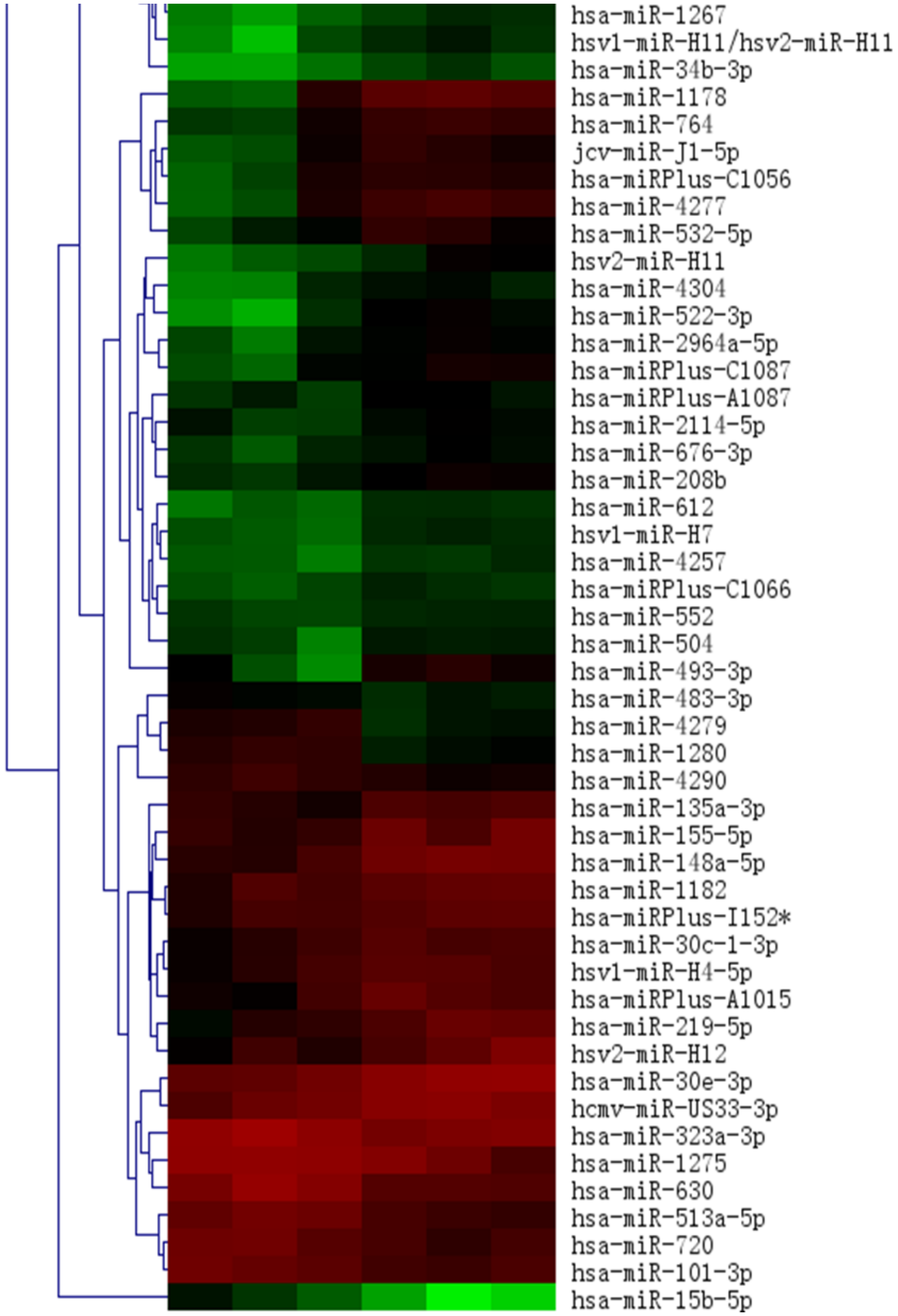
hsa-miR-1267
hsv1-miR-H11/hsv2-miR-H11
hsa-miR-34b-3p
hsa-miR-1178
hsa-miR-764
jcv-miR-J1-5p
hsa-miRPlus-C1056
hsa-miR-4277
hsa-miR-532-5p
hsv2-miR-H11
hsa-miR-4304
hsa-miR-522-3p
hsa-miR-2964a-5p
hsa-miRPlus-C1087
hsa-miRPlus-A1087
hsa-miR-2114-5p
hsa-miR-676-3p
hsa-miR-208b
hsa-miR-612
hsv1-miR-H7
hsa-miR-4257
hsa-miRPlus-C1066
hsa-miR-552
hsa-miR-504
hsa-miR-493-3p
hsa-miR-483-3p
hsa-miR-4279
hsa-miR-1280
hsa-miR-4290
hsa-miR-135a-3p
hsa-miR-155-5p
hsa-miR-148a-5p
hsa-miR-1182
hsa-miRPlus-I152*
hsa-miR-30c-1-3p
hsv1-miR-H4-5p
hsa-miRPlus-A1015
hsa-miR-219-5p
hsv2-miR-H12
hsa-miR-30e-3p
hcmv-miR-US33-3p
hsa-miR-323a-3p
hsa-miR-1275
hsa-miR-630
hsa-miR-513a-5p
hsa-miR-720
hsa-miR-101-3p
hsa-miR-15b-5p
