# Supplementary material for: Comparison of microRNA expression profiles of Kashin-Beck disease, osteoarthritis and rheumatoid arthritis
Source: Sci Rep. 2017 Apr 3;7:540. doi: 10.1038/s41598-017-00522-z (PMC5428653; doi:10.1038/s41598-017-00522-z)
Supplement: Supplementary file 2 — List of target genes of miRNAs differently expressed in KBD vs. Control [file 41598_2017_522_MOESM2_ESM.pdf]

Comparison of microRNA expression profiles of Kashin-Beck disease, osteoarthritis and rheumatoid arthritis

Wenhong Wu[#1], Awen He[#1], Yan Wen[1], Xiao Xiao[1], Jingcan Hao[1], Feng Zhang[1*], Xiong Guo[1*]

Table S1. List of target genes of miRNAs differently expressed in KBD vs. Control

Gene Name	Genebank_ID	Gene Description
PDHA1	NM_000284	pyruvate dehydrogenase (lipoamide) alpha 1
GUCA1A	NM_000409	guanylate cyclase activator 1A (retina)
CYP1A1	NM_000499	cytochrome P450, family 1, subfamily A, polypeptide 1
RHO	NM_000539	rhodopsin
CD4	NM_000616	CD4 molecule
CD79B	NM_000626	CD79b molecule, immunoglobulin-associated beta
CACNA1C	NM_000719	calcium channel, voltage-dependent, L type, alpha 1C subunit
CHRM1	NM_000738	cholinergic receptor, muscarinic 1
CHRNB2	NM_000748	cholinergic receptor, nicotinic, beta 2 (neuronal)
CYP4A11	NM_000778	cytochrome P450, family 4, subfamily A, polypeptide 11
C18ORF1	NM_001003674	chromosome 18 open reading frame 1
BUB3	NM_001007793	budding uninhibited by benzimidazoles 3 homolog (yeast)
C1ORF130	NM_001010980	chromosome 1 open reading frame 130
DCTD	NM_001012732	dCMP deaminase
SORBS3	NM_001018003	sorbin and SH3 domain containing 3
AP2M1	NM_001025205	adaptor-related protein complex 2, mu 1 subunit
CBLN3	NM_001039771	cerebellin 3 precursor
NRF1	NM_001040110	nuclear respiratory factor 1
LRRCC1	NM_001077501	leucine rich repeat and coiled-coil domain containing 1
MED24	NM_001079518	mediator complex subunit 24
UNC13A	NM_001080421	unc-13 homolog A
HDGF	NM_001126050	hepatoma-derived growth factor
LY6E	NM_001127213	lymphocyte antigen 6 complex, locus E
SPRED2	NM_001128210	sprouty-related, EVH1 domain containing 2
GFI1B	NM_001135031	growth factor independent 1B transcription repressor
C8ORF40	NM_001135674	chromosome 8 open reading frame 40
RCC2	NM_001136204	regulator of chromosome condensation 2
SLCO2B1	NM_001145211	solute carrier organic anion transporter family, member 2B1
PFKFB3	NM_001145443	6-phosphofructo-2-kinase/fructose-2,6-biphosphatase 3
SEMA7A	NM_001146029	semaphorin 7A, GPI membrane anchor
LEPRE1	NM_001146289	leucine proline-enriched proteoglycan (leprecan) 1
APAF1	NM_001160	apoptotic peptidase activating factor 1
PLEKHA4	NM_001161354	pleckstrin homology domain containing, family A, member 4
CHAD	NM_001267	chondroadherin
INPPL1	NM_001567	inositol polyphosphate phosphatase-like 1
BSG	NM_001728	basigin (Ok blood group)
CUX1	NM_001913	cut-like homeobox 1
EYA3	NM_001990	eyes absent homolog 3 (Drosophila)
NTSR1	NM_002531	neurotensin receptor 1 (high affinity)
PIGR	NM_002644	polymeric immunoglobulin receptor
RBMS1	NM_002897	RNA binding motif, single stranded interacting protein 1
SELP	NM_003005	selectin P (granule membrane protein 140kDa, antigen

		CD62)
SYT5	NM_003180	synaptotagmin V
VIPR2	NM_003382	vasoactive intestinal peptide receptor 2
CLIP2	NM_003388	CAP-GLY domain containing linker protein 2
WNT8B	NM_003393	wingless-type MMTV integration site family, member 8B
FZD5	NM_003468	frizzled homolog 5 (Drosophila)
PDXK	NM_003681	pyridoxal (pyridoxine, vitamin B6) kinase
RGS11	NM_003834	regulator of G-protein signaling 11
PHOX2B	NM_003924	paired-like homeobox 2b
ORC1L	NM_004153	origin recognition complex, subunit 1-like (yeast)
SYT7	NM_004200	synaptotagmin XVII; synaptotagmin VII
SLC22A13	NM_004256	solute carrier family 22 (organic anion transporter), member 13
ARL3	NM_004311	ADP-ribosylation factor-like 3
PTTG1IP	NM_004339	pituitary tumor-transforming 1 interacting protein
FUT8	NM_004480	fucosyltransferase 8 (alpha (1,6) fucosyltransferase)
NEB	NM_004543	nebulin
SF1	NM_004630	splicing factor 1
AP3B2	NM_004644	adaptor-related protein complex 3, beta 2 subunit
PCSK7	NM_004716	proprotein convertase subtilisin/kexin type 7
RAB36	NM_004914	RAB36, member RAS oncogene family
ACTN4	NM_004924	actinin, alpha 4
PRSS16	NM_005865	protease, serine, 16 (thymus)
APC2	NM_005883	adenomatosis polyposis coli 2
SLC1A7	NM_006671	solute carrier family 1 (glutamate transporter), member 7
LAPTM5	NM_006762	lysosomal multispanning membrane protein 5
METAP2	NM_006838	methionyl aminopeptidase 2
C14ORF1	NM_007176	chromosome 14 open reading frame 1
AKAP10	NM_007202	A kinase (PRKA) anchor protein 10
HNRNPH3	NM_012207	heterogeneous nuclear ribonucleoprotein H3 (2H9)
KIN	NM_012311	KIN, antigenic determinant of recA protein homolog (mouse)
SPDEF	NM_012391	SAM pointed domain containing ets transcription factor
C16ORF5	NM_013399	chromosome 16 open reading frame 5
MEA1	NM_014623	male-enhanced antigen 1
LRRC14	NM_014665	leucine rich repeat containing 14
SIPA1L3	NM_015073	signal-induced proliferation-associated 1 like 3
NUP188	NM_015354	nucleoporin 188kDa
DCAF12	NM_015397	WD repeat domain 40A
SOSTDC1	NM_015464	sclerostin domain containing 1
CHD5	NM_015557	chromodomain helicase DNA binding protein 5
LDLRAP1	NM_015627	low density lipoprotein receptor adaptor protein 1
ZNF589	NM_016089	zinc finger protein 589
SGK2	NM_016276	serum/glucocorticoid regulated kinase 2
HOXC13	NM_017410	homeobox C13
TMEM104	NM_017728	transmembrane protein 104
MKS1	NM_017777	Meckel syndrome, type 1
PNPO	NM_018129	pyridoxamine 5'-phosphate oxidase

USP40	NM_018218	ubiquitin specific peptidase 40
SH3GLB2	NM_020145	SH3-domain GRB2-like endophilin B2
PITPNM2	NM_020845	phosphatidylinositol transfer protein, membrane-associated 2
KAT2A	NM_021078	K(lysine) acetyltransferase 2A
ALOXE3	NM_021628	arachidonate lipoxygenase 3
MS4A1	NM_021950	membrane-spanning 4-domains, subfamily A, member 1
HIF3A	NM_022462	hypoxia inducible factor 3, alpha subunit
KLHL25	NM_022480	kelch-like 25 (Drosophila)
FAM160B2	NM_022749	family with sequence similarity 160, member B2
C8ORF33	NM_023080	chromosome 8 open reading frame 33
GDAP1L1	NM_024034	ganglioside-induced differentiation-associated protein 1-like 1
CAMKV	NM_024046	CaM kinase-like vesicle-associated
ABHD8	NM_024527	abhydrolase domain containing 8
MAP7D3	NM_024597	MAP7 domain containing 3
FAM49A	NM_030797	family with sequence similarity 49, member A
KLF16	NM_031918	Kruppel-like factor 16
FGFBP2	NM_031950	fibroblast growth factor binding protein 2
MXRA8	NM_032348	matrix-remodelling associated 8
LZTS2	NM_032429	leucine zipper, putative tumor suppressor 2
CAPS2	NM_032606	calcyphosine 2
FHDC1	NM_033393	FH2 domain containing 1
CCDC97	NM_052848	coiled-coil domain containing 97
APOA5	NM_052968	apolipoprotein A-V
SMC1B	NM_148674	structural maintenance of chromosomes 1B
PAQR4	NM_152341	progestin and adipoQ receptor family member IV
C19ORF25	NM_152482	chromosome 19 open reading frame 25
GJB4	NM_153212	gap junction protein, beta 4, 30.3kDa
C19ORF21	NM_173481	chromosome 19 open reading frame 21
C17ORF78	NM_173625	chromosome 17 open reading frame 78
ZCCHC12	NM_173798	zinc finger, CCHC domain containing 12
ARNT	NM_178427	aryl hydrocarbon receptor nuclear translocator
BCDIN3D	NM_181708	BCDIN3 domain containing
SPATA12	NM_181727	spermatogenesis associated 12
BCL9L	NM_182557	B-cell CLL/lymphoma 9-like
VSX2	NM_182894	visual system homeobox 2
P4HA3	NM_182904	prolyl 4-hydroxylase, alpha polypeptide III
UBL4B	NM_203412	ubiquitin-like 4B
TEX19	NM_207459	testis expressed 19
